# A dynamical quantum Cheshire Cat effect and implications for counterfactual communication

**DOI:** 10.1038/s41467-021-24933-9

**Published:** 2021-08-06

**Authors:** Yakir Aharonov, Eliahu Cohen, Sandu Popescu

**Affiliations:** 1 School of Physics and Astronomy, Tel Aviv University, Tel Aviv, Israel; 2 Schmid College of Science and Technology and Institute for Quantum Studies, Chapman University, Orange, CA USA; 3 Faculty of Engineering and the Institute of Nanotechnology and Advanced Materials, Bar Ilan University, Ramat Gan, Israel; 4 H. H. Wills Physics Laboratory, University of Bristol, Bristol, UK

**Keywords:** Quantum information, Quantum mechanics

## Abstract

Here we report a type of dynamic effect that is at the core of the so called “counterfactual computation” and especially “counterfactual communication” quantum effects that have generated a lot of interest recently. The basic feature of these counterfactual setups is the fact that particles seem to be affected by actions that take place in locations where they never (more precisely, only with infinitesimally small probability) enter. Specifically, the communication/computation takes place without the quantum particles that are supposed to be the information carriers travelling through the communication channel or entering the logic gates of the computer. Here we show that something far more subtle is taking place: It is not necessary for the particle to enter the region where the controlling action takes place; it is enough for the controlled property of the particle, (i.e., the property that is being controlled by actions in the control region), to enter that region. The presence of the controlled property, without the particle itself, is possible via a quantum Cheshire Cat type effect in which a property can be disembodied from the particle that possesses it. At the same time, we generalize the quantum Cheshire Cat effect to dynamical settings, in which the property that is “disembodied” from the particle possessing it propagates in space, and leads to a flux of “disembodied” conserved quantities.

## Abstract (web summary)

In quantum mechanics, counterfactual behaviours are generally associated with particles being affected by events taking place where they can’t be found. Here, the authors consider extended quantum Cheshire cat scenarios where a particle can be influenced in regions where only its disembodied property has entered.

## Introduction

Quantum mechanics is a notoriously counterintuitive theory. More than ninety years after its basic laws were discovered, there is a widespread agreement that we do not yet really understand what is going on. Nevertheless, in recent years considerable progress has been made, especially in understanding entanglement and Bell-type non-locality. However, the focus of that research is essentially kinematic—these effects depend only on the Hilbert space structure of the set of quantum states. On the other hand, the fundamental dynamic aspects of the theory have been far less investigated, though, undoubtedly, they are equally rich, if not richer. Here we describe a “Dynamic Quantum Cheshire Cat effect”.

In the original Quantum Cheshire Cat effect^1^, it was shown that physical properties can be disembodied from the objects to which they belong. For example, we may find an electron in one location and its spin in a different location. The original effect is however essentially kinematic. Here we show that the disembodied property has a dynamics of its own. Once disembodied from the particle to which it belongs, it can be subsequently affected by external actions even though the particle is not present. In particular, we describe the spatial propagation of such a property, specifically a flux of spin without its corresponding particle.

The motivation that led us to this effect is the desire to better understand “counterfactual computation”^2–8^ and especially “counterfactual communication” quantum effects^9–24^. These are information processing tasks with a very strange property: the particles that are supposed to be the information carriers seem to not actually enter the information processing devices. The starting point of this class of phenomena was the discovery by Elitzur and Vaidman of the so-called “interaction-free measurement”^25^, arguably one of the most striking effects in quantum physics. The basic set-up consists of an object—a “bomb” in the original example—that is ultra-sensitive to photons. Whenever a photon impinges on it, the bomb explodes. What Elitzur and Vaidman showed is that the bomb could nevertheless be investigated with photons without triggering it, if photons are prepared in a coherent superposition of impinging and not impinging on it.

Subsequent work^26^ refined the original interaction-free measurement protocol, which had a failure probability (the bomb exploding) of 50% and raised the probability of success infinitesimally close to 1.

The ideas behind the interaction-free measurements have then been used to design information-processing protocols such as computation and communication, having the same basic characteristic, namely particles being affected by what happens in regions where they do not enter, similar to the probe in the interaction-free measurement. Ever since the discovery of interaction-free measurement and of the subsequent counterfactual information processing protocols there has been an intensive effort to understand what is behind these very puzzling effects.

In the present paper, inspired by the above, we construct a different set-up, that leads us to formulate the dynamic quantum Cheshire Cat effect. In this effect, a physical property can be disembodied from the particle to which it belongs, and can be subsequently affected by external actions even though the particle is not present. Clearly, this could be the key for understanding what happens in counterfactual information processing effects, where information can be accumulated, processed, stored and communicated without particles being present. We believe that in fact, this is the core of all such effects.

The main body of the paper is focused on the dynamic quantum Cheshire Cat effect itself, since it is important and interesting in itself, and its implications are likely to extend well beyond the counterfactual effects that motivated this research. The connection with counterfactual communication will be discussed in the “Discussion” section.

## Results

### The set-up

**Fig. 1:**
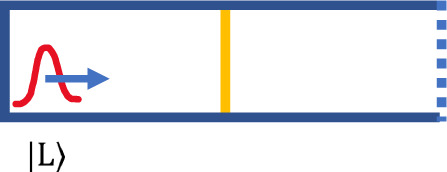
The set-up. A spin 1/2 particle in a box with a completely reflective wall on the left, a spin-independent, partially transparent partition in the middle with, very small transmissivity, and a spin-dependent wall on the right, which is totally reflective for spin $\left|{\uparrow }_{z}\right\rangle$ and totally transparent for $\left|{\downarrow }_{z}\right\rangle$. The particle starts in a wavepacket $\left|L\right\rangle$, next to the left wall, moving right and with spin $\left|{\uparrow }_{z}\right\rangle$.

The experiment at the core of our paper is deceptively simple. We will be interested in some particular cases of the following set-up, illustrated in Fig. 1. Consider a spin 1/2 particle in a box of length *D*. The left wall of the box is completely reflective; the right wall is spin-dependent: it is completely transparent for $\left|{\uparrow }_{z}\right\rangle$ and it completely reflects $\left|{\downarrow }_{z}\right\rangle$. For example, we can take the interaction Hamiltonian between the particle and the spin-dependent wall to be

1 $${\hat{H}}_{{{{{\mathrm{int}}}}}}=\frac{1-{\sigma }_{z}}{2}{V}_{0}\delta (x-{x}_{R})$$

where *x* denotes the location of the particle, *x*_*R*_ is the location of the right wall, and *V*_0_ is very large, essentially infinite. This can be arranged physically by, say, having inside the wall a strong magnetic field along the *z*-axis, plus some other, constant, non-spin dependent, potential. In addition, the box is divided in the middle by a spin-independent partition wall through which the particle can tunnel, but whose transmission coefficient will be taken to be very small.

Suppose the particle is prepared initially in a small wavepacket moving with velocity *v*. The particle will move back and forth in the box, colliding with the box walls and the partition, and leaking out of the box via the right wall. For simplicity, we take the particle to have a large mass, and the velocity *v* high enough, so that during the entire duration of the experiment the spread of the wavepacket is negligible.

For the partition, we take the reflection and transmission amplitudes to be $\cos \epsilon$ and $\sin \epsilon$ respectively, with *ϵ* = *π*/2*N* and *N* integer. Eventually, we will take *ϵ* to be very small (*N* to be large).

### The experiment

We are interested in what happens in the particular case when we start with the particle next to the left wall, moving towards the partition, a state that we denote $\left|L\right\rangle$, and being polarized $\left|{\uparrow }_{z}\right\rangle$. We are interested in what happens by time *t* = 2*NT*, where *T* is the time required to move the distance *D*, the length of the box, (which is also the time required to bounce once back and forth inside half of the box).

To start with, we note that $\left|{\uparrow }_{z}\right\rangle$ is a constant of motion: the only place where there is any spin-dependent interaction is at the right wall, and $\left|{\uparrow }_{z}\right\rangle$ is an eigenstate of that interaction. Hence the particle’s spin remains $\left|{\uparrow }_{z}\right\rangle$ for all times. In this situation, the right wall is completely transparent for the particle.

**Fig. 2:**
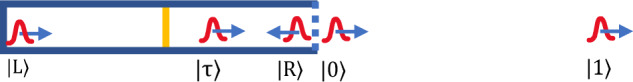
States of interest. Various wavepackets mentioned in the experiment. The arrows denote the direction of movement of the wavepackets. These are various wavepackets that may result at various stages, when a particle starts in an initial wavepacket, $\left|L\right\rangle$ next to the left wall, moving towards right, and then is reflected by, or tunnels through, a semi-transparent partition in the middle or a spin-dependent wall at the right end of the box. $\left|R\right\rangle$ is a wavepacket inside the box, next to the right wall, moving towards left and $\left|0\right\rangle$ is a wavepacket next to the right wall but outside the box, moving to the right. The wavepacket $\left|1\right\rangle$ is a distance *D*, equal to the length of the box, away from the right wall; it is here where the wavepacket $\left|0\right\rangle$ will get after a time *T* = *D*/*v*. Other states $\left|2\right\rangle$, $\left|3\right\rangle$,... are situated further away, to the right, at intervals *D* from one another and are not illustrated in the figure. The wavepacket $\left|\tau \right\rangle$ represents a particle localized in the right half of the box, moving towards right, at a distance *vτ* from the left wall, with *T*/2 ≤ *τ* < *T*. Note that not all these wavepackets are simultaneously present during the experiment. Also, the spin degree of freedom, and the normalization with which various wavepackets may appear at various times in the experiment are not marked in the figure.

The time evolution can easily be solved. It is convenient to consider the state of the particle at times *t*_*n*_ = *nT* with *n* a positive integer. Apart from the state $\left|L\right\rangle$ defined above, other states that will be of interest for us are $\left|k\right\rangle$, with *k* a positive integer, which denotes the wavepacket situated outside the box, at a distance *kD* from its right end, moving away from the box. In particular, the state $\left|0\right\rangle$ means the particle is just outside the right wall (see Fig. 2).

Let $\hat{U}$ denote the time evolution operator for a time *T*. We have

2 $$	\hat{U}\big|L\big\rangle \left|{\uparrow }_{z}\right\rangle =\cos \epsilon \big|L\big\rangle \left|{\uparrow }_{z}\right\rangle +\sin \epsilon \big|0\big\rangle \left|{\uparrow }_{z}\right\rangle \\ 	 \hat{U}\big|k\big\rangle \left|{\uparrow }_{z}\right\rangle =\left|(k+1)\right\rangle \left|{\uparrow }_{z}\right\rangle$$

What happens is that when the particle starts in the state $\left|L\right\rangle$, next to the left wall, it moves towards the partition and collides with it at time *T*/2. As a result of this collision, with amplitude $\cos \epsilon$, the wavepacket is reflected, moves back towards the left wall, and at time *T* collides with it and is reflected, ending in the state $\left|L\right\rangle$, as it was initially, but with corresponding reduced amplitude, while with amplitude $\sin \epsilon$, the wavepacket is transmitted through the partition and at time *T* it emerges from the box, ending in state $\left|0\right\rangle$ with the corresponding reduced amplitude. On the other hand, if the particle is already outside the box, in state $\left|k\right\rangle$, then after time *T* it evolves to $\left|(k+1)\right\rangle$, a state further away from the box by the distance *D*. The operator $\hat{U}$ describes this entire process.

It is now trivial to calculate what happens at time *nT*, with 1 ≤ *n*, when $\left|\Psi (0)\right\rangle =\big|L\big\rangle \left|{\uparrow }_{z}\right\rangle$. The state becomes

3 $$\begin{array}{ll}&\big|\Psi (nT)\big\rangle ={\hat{U}}^{n}\big|L\big\rangle \left|{\uparrow }_{z}\right\rangle \\ =&\kern-0.5pc{\cos }^{n}\epsilon \big|L\big\rangle \left|{\uparrow }_{z}\right\rangle +\mathop{\sum }\limits_{k=0}^{n-1}\sin \epsilon {\cos }^{k}\epsilon \left|n-1-k\right\rangle \left|{\uparrow }_{z}\right\rangle \end{array}$$

After every collision with the partition, the wavepacket in the left side of the box loses a fraction of its amplitude, generating a wavepacket that tunnels to the right through the partition, and which then goes out of the box. Eventually, the particle will leak out of the box almost entirely, but this takes a long time, of order of *N*^2^*T*.

Importantly for us, however, in the limit of small transmissivity (large *N*), for times of order *NT* the particle is essentially still on the left side, leaking out of the box only with an infinitesimal probability of order *O*(1/*N*), i.e.,

4 $$\left|\Psi (2NT)\right\rangle ={\hat{U}}^{2N}\big|L\big\rangle \left|{\uparrow }_{z}\right\rangle =\big|L\big\rangle \left|{\uparrow }_{z}\right\rangle +\left|O(1/N)\right\rangle$$

where $\left|O(1/N)\right\rangle$ denotes corrections of order 1/*N*.

We can see this by directly calculating the probability of remaining on the left side, ${\cos }^{2N}\epsilon ={\cos }^{2N}(\pi /2N)=1-O(1/N)$ but it is actually illuminating to calculate instead the probability of leaving the box. The important feature is that the tunnelled wavepackets do not overlap with each other so when determining the total probability of leaving they add up in probability, not in amplitude

5 $$\mathop{\sum }\limits_{k=0}^{N-1}{(\sin \epsilon {\cos }^{k}\epsilon )}^{2}\le \mathop{\sum }\limits_{k=0}^{N-1}{\sin }^{2}\epsilon \approx N{\epsilon }^{2}=\frac{\pi }{2}\epsilon =\frac{{\pi }^{2}}{4N}$$

which goes to zero in the limit of small transmissivity (large *N*).

### The paradox

The situation seems completely trivial: the particle bounces back and forth in the left half of the box, leaking infinitesimally small wavepackets via tunnelling, and its spin remains undisturbed, $\left|{\uparrow }_{z}\right\rangle$. Yet, at a closer look, we see that something quite interesting happens.

Consider the spin along *x*. Since the particle is prepared polarized in the *z-*direction, the *x* component is undefined: it is an equal superposition of $\left|{\uparrow }_{x}\right\rangle$ and $\left|{\downarrow }_{x}\right\rangle$, namely $\left|{\uparrow }_{z}\right\rangle =\frac{1}{\sqrt{2}}\big(\left|{\uparrow }_{x}\right\rangle +\left|{\downarrow }_{x}\right\rangle \big)$. Furthermore, since $\left|{\uparrow }_{z}\right\rangle$ is a constant of motion, this superposition of $\left|{\uparrow }_{x}\right\rangle$ and $\left|{\downarrow }_{x}\right\rangle$, remains the same at all times. However, each component changes with time. Specifically, as we will show, if at time 2*NT* we find the particle in the left half of the box—which we can arrange to happen with probability as close to 1 as we desire—the spin along the *x-*axis flips: $\left|{\uparrow }_{x}\right\rangle \to \left|{\downarrow }_{x}\right\rangle$ and $\left|{\downarrow }_{x}\right\rangle \to \left|{\uparrow }_{x}\right\rangle$.

Another way to look at the situation is to note that when we look at the *x*-spin observable, i.e., at the *σ*_*x*_ operator, its Heisenberg equation of motion tells us that if we find the particle in the left half of the box then *σ*_*x*_(*t* = 2*NT*) = −*σ*_*x*_(*t* = 0) (see the proof in the next section).

The fact that there are situations in which the quantum state of a system does not change but nevertheless some observables change is actually extremely common, though, as far as we know, the very existence of this effect and its implications are not widely appreciated (for various discussions of this see refs. ^27–29^ where the notion of two-time observables was introduced and analyzed, and^30–34^ where the disturbance due to measurements and its implications for the Heisenberg uncertainties are studied). This situation occurs every time when a particle is in an eigenstate of a non-zero Hamiltonian. This is not what is interesting in our example. What is interesting is how this happens in our case.

To put things in perspective, a trivial example is that of a spin 1/2 particle prepared in the state $\left|{\uparrow }_{z}\right\rangle$ and placed in a magnetic field oriented along the *z*-axis. In this case, the state is an eigenstate of the Hamiltonian and remains unchanged (up to an unobservable overall phase factor), but all the spin components in the plane orthogonal to *z* undergo Larmor precession, which we see if we solve their Heisenberg equations of motion. If we were to measure their values at any time, the outcomes of the measurement would be completely random, but if we look at the correlations between their values at different moments in time, we find that they change.

The above case is trivial, and we understand very well why these spin components change: the magnetic field acts upon them. And here lies our paradox: if at the end of the experiment we find the particle in the left-side of the box, the particle must have stayed there for the entire duration of the experiment. But there is no magnetic field there—the only place where there is a magnetic field is in the spin-dependent wall, at the right-end of the box. Indeed, up to terms whose total magnitude we can make as small as we want by decreasing the transmissivity of the partition, the wavefunction remained localized in the left-side of the box for the entire duration of the experiment where there is no magnetic field. Moreover, even the infinitesimally small wave-packets that leaked out of the left-side of the box and reached the spin-dependent wall on the right could not contribute to what is happening on the left-side, as for them the spin-dependent wall is fully transparent, so they exit the box and never return. Nevertheless, the Heisenberg equations of motion tell that the *x*-component of the spin changes.

Even more surprising, looking solely at the solution of the Schrödinger equation, the situation in our experiment seems to be identical to the case in which there is no wall at all closing the right end of the box. Indeed, when initially the particle is prepared $\left|{\uparrow }_{z}\right\rangle$ the solutions of the Schrödinger equation with spin-dependent wall or no wall at all are identical, as the particle simply does not see the wall even if the wall is there. Yet, when the wall is present the *x*-spin component flips, while when there is no wall, it does not.

### The time evolution of *σ*_*x*_

In the previous section we simply claimed that by time 2*NT* the *x*-spin component flips; we will now prove it. Rather than attempting to calculate the evolution of *σ*_*x*_ by solving the Heisenberg equations of motion directly, it is simpler to go via an indirect route. Consider first what happens if instead of starting with the initial state $\big|L\big\rangle \left|{\uparrow }_{z}\right\rangle$ we would start with $\left|\Phi (0)\right\rangle =\big|L\big\rangle \left|{\downarrow }_{z}\right\rangle$. In this case, the particle sees the box closed on the right by a completely reflecting wall, hence the box is now closed at the two ends.

With $\left|R\right\rangle$ denoting the wavepacket inside the box, next to the right wall, and moving to the left, towards the partition, we have

6 $$\hat{U}\big|L\big\rangle \left|{\downarrow }_{z}\right\rangle =\cos \epsilon \big|L\big\rangle \left|{\downarrow }_{z}\right\rangle +\sin \epsilon \big|R\big\rangle \left|{\downarrow }_{z}\right\rangle$$

7 $$\hat{U}\big|R\big\rangle \left|{\downarrow }_{z}\right\rangle =\cos \epsilon \big|R\big\rangle \left|{\downarrow }_{z}\right\rangle -\sin \epsilon \big|L\big\rangle \left|{\downarrow }_{z}\right\rangle .$$

What happens is that the particle, starting in state $\left|L\right\rangle$, next to the left wall and moving towards the centre, traverses the left side of the box, and hits the partition at time *T*/2. Then it gets partially reflected by the partition, with amplitude $\cos \epsilon$, traverses back the left side of the box collides with the left wall and at time *T* it gets again in state $\left|L\right\rangle$, with the reduced amplitude $\cos \epsilon$, while with amplitude $\sin \epsilon$ the particle tunnels through the partition, traverses the right side of the box and gets reflected by the right wall, ending at time *T* in state $\left|R\right\rangle$ with the corresponding amplitude, $\sin \epsilon$. A similar thing happens when starting in the state $\left|R\right\rangle$. The operator $\hat{U}$ describes this whole evolution.

The evolution for a time *nT* leads to

8 $${\hat{U}}^{n}\big|L\big\rangle \left|{\downarrow }_{z}\right\rangle =\cos (n\epsilon )\big|L\big\rangle \left|{\downarrow }_{z}\right\rangle +\sin (n\epsilon )\big|R\big\rangle \left|{\downarrow }_{z}\right\rangle .$$

9 $${\hat{U}}^{n}\big|R\big\rangle \left|{\downarrow }_{z}\right\rangle =\cos (n\epsilon )\big|R\big\rangle \left|{\downarrow }_{z}\right\rangle -\sin (n\epsilon )\big|L\big\rangle \left|{\downarrow }_{z}\right\rangle .$$

In particular, we see that starting in the state $\left|L\right\rangle \left|{\downarrow }_{z}\right\rangle$ the particle oscillates between the left and right side of the box, with period *T*_osc_ = 4*NT* = 2*πT*/*ϵ*. We are interested in what happens at time 2*NT*, at half the period. By this time the particle would have first transitioned to the right side of the box, in state $\left|R\right\rangle$, where it got at time *NT* and then, at *t* = 2*NT*, the particle is again in the left side, where it started, but in state $-\left|L\right\rangle$, the state has acquired a negative sign. In other words,

10 $${\hat{U}}^{2N}\big|L\big\rangle \left|{\downarrow }_{z}\right\rangle =-\big|L\big\rangle \left|{\downarrow }_{z}\right\rangle$$

Suppose now that we start with the state $\left|L\right\rangle \left|{\uparrow }_{x}\right\rangle$. We then have

11 $${\hat{U}}^{2N}\big|L\big\rangle \left|{\uparrow }_{x}\right\rangle 	=\displaystyle{\hat{U}}^{2N}\big|L\big\rangle \frac{1}{\sqrt{2}}\big(\left|{\uparrow }_{z}\right\rangle +\left|{\downarrow }_{z}\right\rangle \big) \\ 	 = \displaystyle\big|L\big\rangle \frac{1}{\sqrt{2}}\big(\left|{\uparrow }_{z}\right\rangle -\left|{\downarrow }_{z}\right\rangle \big)+\left|O(1/N)\right\rangle \\ 	 =\displaystyle\big|L\big\rangle \left|{\downarrow }_{x}\right\rangle +\left|O(1/N)\right\rangle .$$

That is, if we start with spin $\left|{\uparrow }_{x}\right\rangle$, after time 2*N* it flips to $\left|{\downarrow }_{x}\right\rangle$, up to corrections of *O*(1/*N*). Similarly,

12 $${\hat{U}}^{2N}\big|L\big\rangle \left|{\downarrow }_{x}\right\rangle =\big|L\big\rangle \left|{\uparrow }_{x}\right\rangle +\left|O(1/N)\right\rangle .$$

Hence we can now obtain the Heisenberg evolution:

13 $${\hat{U}}^{{{{\dagger}}} 2N}\big|L\big\rangle \big\langle L\big|{\sigma }_{x}{\hat{U}}^{2N}=\big|L\big\rangle \big\langle L\big|(-{\sigma }_{x})+O(1/N).$$

On the other hand, when there is no wall closing the right side of the box, the evolution of $\left|{\uparrow }_{z}\right\rangle$ and $\left|{\downarrow }_{z}\right\rangle$ is identical, so they accumulate no phase difference; hence their superposition $\left|{\uparrow }_{x}\right\rangle =\frac{1}{\sqrt{2}}\big(\left|{\uparrow }_{z}\right\rangle +\left|{\downarrow }_{z}\right\rangle \big)$ remains $\left|{\uparrow }_{x}\right\rangle$ all the time, i.e., *σ*_*x*_ does not flip.

### Observational consequences

As discussed in the above sections, the Heisenberg equations of motion show that when at time *t* = 2*NT* we find the particle in the left half of the box—which is almost always when the transmission coefficient *ϵ* is infinitesimally small—the spin component in the *x-*direction flips from what it was at the start of the experiment. In other words, the correlation between the initial and final values of the *x*-spin components changes due to the presence of the spin-dependent wall. But that was a purely mathematical analysis—no measurements were considered that could verify the effect. We will do that now. The challenge is to observe this without disrupting the original set-up.

Of course, if we attempt to start by measuring *σ*_*x*_ at *t* = 0, in order to see how it changes by *t* = 2*NT*, we disturb the initial state $\left|{\uparrow }_{z}\right\rangle$ and generate a $\left|{\downarrow }_{z}\right\rangle$ component. This component then propagates up to the spin-dependent wall and bounces back from there, ending back on the left side at *t* = 2*NT*, so there would be no surprise that the particle’s spin feels the magnetic field. To avoid this, we start by measuring *σ*_*x*_ “weakly”, i.e., in a manner that will produce as small disturbance as we desire. Of course, such a measurement cannot be precise, but repeating the experiment many times, we can extract the desired information. (A similar procedure, in the context of Heisenberg’s measurement−disturbance relation, was considered in ref. ^33^ and was experimentally implemented in ref. ^34^).

To this end, we use a measuring device with a pointer $\hat{q}$, prepared in the initial state (up to normalization)

14 $$\phi (q)={{{{{\mathrm{e}}}}}}^{-\frac{{q}^{2}}{4{\Delta }^{2}}},$$

where Δ determines the uncertainty in the initial position of the pointer. We couple this test particle to our spin by the interaction Hamiltonian

15 $${\hat{H}}_{{{{{\mathrm{test}}}}}}=\lambda \delta (t)\hat{p}{\sigma }_{x}.$$

where $\hat{p}$ is the momentum conjugate to $\hat{q}$ and *λ* is a numerical constant, which has the role of a coupling constant. We can then make the disturbance as small as we want by taking *λ* appropriately small.

The time evolution operator that describes the measuring interaction is

16 $$\hat{V}={{{{{\mathrm{e}}}}}}^{-\frac{{{{{\mathrm{i}}}}}}{\hslash }\lambda \hat{p}{\sigma }_{x}}$$

which is the shift operator that shifts *q* by the value *λσ*_*x*_. In other words, the pointer $\hat{q}$ moves proportionally to the value of *σ*_*x*_, with proportionality constant *λ*.

After measuring *σ*_*x*_ in this weakly disturbing way at *t* = 0, just after we prepared the initial state, we let the particle and measuring device evolve until *t* = 2*NT*. At that moment their state is

17 $${\hat{U}}^{2N}\hat{V}\left|{\uparrow }_{z}\right\rangle \big|L\big\rangle {{{{{\mathrm{e}}}}}}^{-\frac{{q}^{2}}{4{\Delta }^{2}}}.$$

We then check to see if it is on the left side of the box and perform there an ideal (strong) measurement of *σ*_*x*_. We are interested to see how the *σ*_*x*_ values measured at *t* = 0 and *t* = 2*NT* correlate.

When the final measurement finds *σ*_*x*_ = +1, the state of the pointer *q*, used for measuring *σ*_*x*_ at *t* = 0 is

18 $$\big\langle L\big|\left\langle {\uparrow }_{x}\right|{\hat{U}}^{2N}\hat{V}\left|{\uparrow }_{z}\right\rangle \big|L\big\rangle {{{{{\mathrm{e}}}}}}^{-\frac{{q}^{2}}{4{\Delta }^{2}}} = \frac{1}{\sqrt{2}}{{{{{\mathrm{e}}}}}}^{-\frac{{(q+\lambda {\sigma }_{x}^{w})}^{2}}{4{\Delta }^{2}}}+{{{{{{{\mathcal{O}}}}}}}}({\lambda }^{2})$$

where we have used the first-order approximation of $\hat{V}$ in *λ* and where

19 $${\sigma }_{x}^{w}=\displaystyle\frac{\big\langle L\big|\left\langle {\uparrow }_{x}\right|{\hat{U}}^{2N}{\sigma }_{x}\left|{\uparrow }_{z}\right\rangle \big|L\big\rangle }{\big\langle L\big|\left\langle {\uparrow }_{x}\right|{\hat{U}}^{2N}\left|{\uparrow }_{z}\right\rangle \big|L\big\rangle }$$

is the so-called “weak” value of *σ*_*x*_ between the initial state $\left|{\uparrow }_{z}\right\rangle \left|L\right\rangle$ and the final state $\big\langle L\big|\left\langle{\uparrow }_{x}\right|{\hat{U}}^{2N}$ (which is the state corresponding to the result of the final measurement, i.e., that the particle was found at *t* = 2*NT* in the left side of the box, with *σ*_*x*_ = +1, propagated backwards in time to *t* = 0). This is an instance of the general result that the state of the pointer used in a weak measurement of an observable $\hat{A}$ indicates the weak value

20 $${A}^{w}=\displaystyle\frac{\langle {\Psi }_{f}|\hat{W}({t}_{f},{t}_{0})\hat{A}\hat{W}({t}_{0},{t}_{i})|{\Psi }_{i}\rangle }{\langle {\Psi }_{f}|\hat{W}({t}_{f},{t}_{i})|{\Psi }_{i}\rangle }$$

where $|{\Psi }_{i}\rangle$ and $|{\Psi }_{f}\rangle$ are the pre and post-selected states, *t*_*i*_ and *t*_*f*_ are the times of the initial preparation and post-selection respectively, *t*_0_ is the time when the measurement occurred and $\hat{W}$ is the time evolution operator^35^ (see also “Methods”, subsection Weak measurements).

Using Eqs. (11) and (12) we find that

21 $${\sigma }_{x}^{w}=-1+{{{{{{{\mathcal{O}}}}}}}}\Big(\frac{1}{N}\Big).$$

Putting all together, we see that when the measurement performed at *t* = 2*NT* found the spin in the left side of the box, (which happens with probability close to 1) and *σ*_*x*_ = +1, the pointer *q* of the measuring device used to measure the *σ*_*x*_ at *t* = 0 shifted to the value −*λ*. Of course, with *λ* small, the shift in the position of the pointer *q* is smaller than its uncertainty, but repeating the experiment many times we are able to determine the shift with as much precision as desired.

As the pointer $\hat{q}$ moves proportionally to the value of *σ*_*x*_, with proportionality constant *λ*, this means that the pointer indicates that at *t* = 0, the *x*-spin component was *σ*_*x*_ = −1, confirming our claim that the value of *σ*_*x*_ at *t* = 0 is opposite to that at the final time *t* = 2*NT*.

A similar conclusion also holds when at time *t* = 2*NT* we found the spin in the left side of the box but with *σ*_*x*_ = −1.

### The flux of spin without particles

We now come to a very interesting aspect of our problem. Spin is a conserved quantity as long as the particle moves in a region where there is no magnetic field. As we have seen, the *x*-component of the spin in the left half of the box changes, but the only place where there is a magnetic field is in the right end of the box, in the spin-dependent wall. But how does it get there? Recall that the overall probability of the particle to reach the spin-dependent wall can be made as small as we want. Furthermore, even this infinitesimally small amount of cases cannot have any effect on what happens on the left side of the box, since in all the cases when the particle did reach the spin-dependent wall they leave the box and never return to the left side, as the time evolution (3) shows. Hence, if at the end of the experiment we find the particle in the left half of the box, it must have been there all the time.

The answer, we will show, is that there is a flux of spin without particles that carry it, in a version of the “Quantum Cheshire Cat” effect^1^, in which properties can be disembodied from the particle that possesses them, like the grin from the famous cat.

Even more interesting, the spin flux originates from the left half of the box and propagates to the right, and not from the region of the wall towards the particle. In other words, the particle has to originate this flux. But while the particle is in the left half of the box, it has no knowledge whether or not at the right there is a spin-dependent wall or not, which raises the question of how does the particle knows when to originate the flux and when not?

Equation (3), of course, describes the evolution of the particle when no measurement to test it is performed. Again, to observe it, we will perform weak measurements that only infinitesimally disturb the particle, similarly to that described before.

To observe the details of the spin flow, we need to analyze the experiment in more detail. Up to this point, we only looked at times *t* = *nT*, multiples of the period of the bouncing back and forth inside one half of the box. We need now to look at more intermediate times.

Let *U*(*τ*), with 0 ≤ *τ* < *T* represent the time evolution for a time *τ* shorter than the period *T*.

Suppose that at time *t* = *nT* + *τ* we test whether the particle is on the right-hand half of the box, given that we find it on the left side at time *t* = 2*NT*, (which is, as we have shown, almost always). The times of interest are in the second part of each period (i.e., *T*/2 ≤ *τ* < *T*), which is when a tunnelled wavepacket is formed and traverses the right half of the box on its way out.

We already know that the amplitude to find the particle on the right-hand side after the *n*-th collision with the partition is $\sin \epsilon {\cos }^{n}\epsilon$, so very low to start with. But if we further condition it by the fact that at the final time *t* = 2*NT* we find the particle on the left side, we now expect the probability to find it at the intermediate time in the right side should be strictly zero: indeed, given that the spin is $\left|{\uparrow }_{z}\right\rangle$, each tunnelled wavepacket subsequently emerges from the box and never comes back. The only way to find it on the left side at the final time is therefore not to have tunnelled at all. This intuition is confirmed by the weak measurement: if we weakly measure *P*_*R*_, the projector on the right side at any arbitrary time *t* = *nT* + *τ* we find that the pointer indicates zero. Indeed, the pointer will be displaced to the "weak value" of *P*_*R*_ (see Eq. (20))

22 $${\hat{P}}_{R}^{w}=\frac{\big\langle L\big|\left\langle {\uparrow }_{x}\right|{\hat{U}}^{2N-n-1}\hat{U}(T-\tau ){\hat{P}}_{R}\hat{U}(\tau ){\hat{U}}^{n}\left|{\uparrow }_{z}\right\rangle \big|L\big\rangle }{\big\langle L\big|\left\langle {\uparrow }_{x}\right|{\hat{U}}^{2N}\left|{\uparrow }_{z}\right\rangle \big|L\big\rangle }=0.$$

This weak value is equal to zero because ${\hat{P}}_{R}\hat{U}(\tau ){\hat{U}}^{n}\left|{\uparrow }_{z}\right\rangle \big|L\big\rangle$ is a wavepacket localized in the right half of the box, originated from tunnelling during the *n*-th collision and, by the final time *t* = 2*NT* is outside of the box, i.e., up to a normalization factor,

23 $${\hat{U}}^{2N-n-1}\hat{U}(T-\tau ){\hat{P}}_{R}\hat{U}(\tau ){\hat{U}}^{n}\left|{\uparrow }_{z}\right\rangle \big|L\big\rangle =\left|{\uparrow }_{z}\right\rangle \left|N-n\right\rangle$$

and it is orthogonal to $\left|{\uparrow }_{z}\right\rangle \big|L\big\rangle$, hence the numerator in (22) is zero.

(In the above we assumed that the measurement of the spin at the end of the measurement yielded *σ*_*x*_ = 1; the same holds if this measurement yields *σ*_*x*_ = −1.)

Therefore, indeed, in the case that at the end we find the particle in the left half, there is no flux of particles traversing the right half of the box. Yet, its *x*-spin component changes. The spin difference must leak out somehow.

Let us ask now whether there is a spin in the right half of the box. This seems silly, as we have just proven that there are no particles there at any time. Yet, let us ask. If we perform a weak, very non-disruptive measurement of ${\hat{P}}_{R}{\sigma }_{x}$ at time *t* = *nT* + *τ* with *T*/2 ≤ *τ* < *T*, and suppose that at the end of the experiment, a measurement of position and spin of the particle finds the particle in the left side of the box, with spin *σ*_*x*_ = 1, the measurement at *t* = *T* + *τ* yields (see “Methods”, subsection Spin measurements).

24 $$\begin{array}{ll}&\left(\right.{\hat{P}}_{R}{\sigma }_{x}{\left)\right.}^{w} \\ =&\displaystyle\frac{\big\langle L\big|\left\langle {\uparrow }_{x}\right|{\hat{U}}^{2N-n-1}\hat{U}(T-\tau ){\hat{P}}_{R}{\sigma }_{x}\hat{U}(\tau ){\hat{U}}^{n}\left|{\uparrow }_{z}\right\rangle \big|L\big\rangle }{\big\langle L\big|\left\langle {\uparrow }_{x}\right|{\hat{U}}^{2N}\left|{\uparrow }_{z}\right\rangle \big|L\big\rangle }\\ =&\displaystyle-\frac{{\cos }^{n}\epsilon \sin \epsilon \sin (2N-n-1)\epsilon }{{\cos }^{2N}\epsilon }\end{array}$$

which is different from zero, even though there is no particle there.

We can in fact study the evolution in more detail. Instead of just checking for the existence of the spin in the whole right half of the box, we check its precise position and how it changes in time. We can do this by measuring at time *t* = *nT* + *τ* the operator ${\hat{P}}_{R,\tau }{\sigma }_{x}$ where ${\hat{P}}_{R,\tau }$ is the projector on the location in the right half of the box where the tunnelled wavepacket is at time *τ* after the tunnelling occurred. We find that the entire spin in the right half of the box is actually concentrated in this region, i.e.,

25 $$\left(\right.{\hat{P}}_{R,\tau }{\sigma }_{x}{\left)\right.}^{w}=\left(\right.{\hat{P}}_{R}{\sigma }_{x}{\left)\right.}^{w}$$

while if at the same time we measure the spin at other locations in the right half of the box we find zero.

Finally, we can also check for the existence of a particle and spin outside the box. A similar calculation to the one above, taking into account that the propagation of a wavepacket is spin-independent, $\hat{U}\left|k\right\rangle \left|{\uparrow }_{z}\right\rangle =\left|k+1\right\rangle \left|{\uparrow }_{z}\right\rangle$ and $\hat{U}\left|k\right\rangle \left|{\downarrow }_{z}\right\rangle =\left|k+1\right\rangle \left|{\downarrow }_{z}\right\rangle$, shows that, given that at the end of the experiment we find the particle in the left half of the box, there is neither a particle nor a spin outside the box at any time.

We, therefore, see that every period *T,* a pulse of spin propagates from the partition towards the spin-dependent wall and it is absorbed by the wall.

The total spin that left the left half of the box, propagated to the wall and got absorbed there can be found by summing the spin carried in each pulse during the experiment:

26 $$\mathop{\sum }\limits_{n=0}^{2N-1}\frac{{\cos }^{n}\epsilon \sin \epsilon \sin (2N-n-1)\epsilon }{{\cos }^{2N}\epsilon }=-2+{{{{{{{\mathcal{O}}}}}}}}\Big(\frac{1}{N}\Big)$$

(see “Methods”, subsection Spin flux), meaning that the spin in the left half of the box increased by $2+{{{{{{{\mathcal{O}}}}}}}}(\frac{1}{N})$, which is precisely the difference between the initial spin, which we argued is *σ*_*x*_ = −1 and the final spin, which we measured to be *σ*_*x*_ = +1.

## Discussion

In this paper we analyzed a set-up that is at the core of a host of “counterfactual” phenomena. What all these phenomena have in common is that events in a given space region depend on what happens in a different space region (the “control” region) despite the particle having infinitesimally small probability of ever entering that region. What we have shown here is that this apparent paradox can be explained by the fact that it is not important for the particle to enter the control region; it is enough for the controlled property, (i.e., the property that is being controlled by actions in the control region), of the particle to enter that region. The presence of the controlled property, without the particle itself, is possible via a quantum Cheshire Cat effect.

As we mentioned in the “Introduction”, our work was inspired by the desire to better understand the so-called counterfactual information processing protocols. The connection to counterfactual communication is immediate. Imagine the set-up described in our paper, with Alice next to the left-side of the box and Bob next to the right-side. Bob could communicate information to Alice by inserting or not inserting the spin-dependent wall. Say, when Bob wants to communicate a “0” he could leave the right-end of the box open, while when he wants to communicate a “1” he could insert the spin-dependent wall. In her turn, Alice could perform a weak measurement of *σ*_*x*_ at the start of the protocol and a strong measurement of *σ*_*x*_ at the end. In both cases (i.e., when Bob sends a “0” or a “1”) the particle remains at all time next to Alice (except an infinitesimally small number of cases). Yet, by detecting if *σ*_*x*_ flipped or not, Alice can learn the bit sent by Bob. Hence this constitutes counterfactual communication. All of our analysis and insight can now be seen to apply to counterfactual communication. That is, even though the particle which was supposed to be the carrier is not sent through the channel, our results show that the disembodied information carrier, the spin, is in fact sent.

True, the above particular protocol, while exhibiting counterfactual communication, is quite inefficient. Indeed, in one round of the protocol, the weak measurement provides only very little information about the initial value of *σ*_*x*_; to decide whether *σ*_*x*_ flips or not requires the protocol to be repeated many times, so the communication rate is very small. But this protocol is just a simple, direct, and unsophisticated application of our set-up, while efficient counterfactual communication protocols are far more complex. Analyzing those more complex examples in the light of the present insights is left for future work.

It is also interesting to compare the effect present in this paper with the so-called “negative-result measurements”, a class of effects in which in some sense—different from ours—a particle is influenced by actions in a region where the particle is not found^36, 37^. The famous example by Dicke^37^ is that of a particle in the ground state of a box. A measurement of position is made by sending a beam of light through the right half of the box. There is a probability of 50% of finding the particle there. The interesting case is when the light is not scattered, i.e., the particle is not found in the right half of the box. However, although the light is not scattered, the wavefunction of the particle is changed. Before the measurement the wavefunction was the ground state, which is spread on both sides of the box; after the measurement the wavefunction it is collapsed to the left side only. So although the particle was not found on the right side and the light beam was not scattered, the particle gained energy (the post-measurement state is a superposition of ground plus excited states, while initially it was ground state only). The difference between this effect and ours is that in the case of the “negative-result measurements” a significant part of the wavefunction is initially in the interaction region, (i.e., the right side of the box, where the beam of light was sent, in Dicke’s example). Crucially, the size of the effect depends on the overlap of the wavefunction with the interaction region. Had the particle in Dicke’s example been prepared in a state with small overlap with the right side of the box, the state would have been only a little perturbed by the beam of light; in the limit of zero initial overlap, the particle would have not been perturbed at all and the effect vanishes. In our case, the overlap of the wavefunction with the interaction region can be made as close to zero as we want, and the effect remains. This shows the different nature of the two effects.

Finally, in this work, we present a dynamic Cheshire Cat effect. We would like to emphasize that our analysis is not instead of solving the Schrödinger equation, rather, it shows more details of the phenomena involved. In doing so we hope to achieve a better understanding of the true nature of quantum mechanics.

## Methods

### Weak measurements

The weakly disturbing measurement discussed here was first introduced in ref. ^35^. We give the main result here, for convenience.

In order to measure an arbitrary observable $\hat{A}$ at time *t* = *t*_0_ we follow the von Neumann measuring formalism. We consider a measuring device with a pointer $\hat{q}$ prepared in the initial state (up to normalization) $\phi (q)={{{{{\mathrm{e}}}}}}^{-\frac{{q}^{2}}{4{\Delta }^{2}}}$ where Δ is the uncertainty in the initial position of the pointer, and couple the measuring device to the measured system via the interaction Hamiltonian

27 $$\hat{H}=\lambda \delta (t-\tau )\hat{A}\hat{p}$$

where $\hat{p}$ is the momentum canonically conjugate to the pointer position $\hat{q}$. We will be interested in the case in which the coupling constant *λ* is small. In the rest of the time, the measuring device is supposed to remain unchanged, i.e., to have zero Hamiltonian. The unitary evolution corresponding to the interaction is

28 $$\hat{V}={{{{{\mathrm{e}}}}}}^{-{i}\,\lambda\, \hat{A}\,\hat{p}}$$

which is the shift operator, which shifts the pointer by the value of $\hat{A}$.

Here we are interested in performing the measurement on a system that was prepared at the initial time *t* = *t*_*i*_ in the initial state $\left|{\Psi }_{i}\right\rangle$ and which, except for *t* = *t*_0_ when the measurement is performed evolves under the time evolution operator $\hat{W}$. Furthermore, we are interested in the result of this weak measurement in the case in which a second, later measurement, of some other operator *B*, taking place at *t* = *t*_*f*_ happened to yield the eigenvalue corresponding to its eigenstate $|{\Psi }_{f}\rangle$.

The system evolves from *t* = *t*_*i*_ to *t* = *t*_0_ under the evolution operator $\hat{W}({t}_{0},{t}_{i})$. Then, the state of the system and measuring device immediately after the measurement is given by

29 $$\displaystyle{{{{{\rm{e}}}}}}^{-{{i}}\uplambda \hat{{A}}\hat{{p}}}\hat{W}({t}_{0},{t}_{i})\left|{\Psi }_{i}\right\rangle \left|{{{{{\mathrm{e}}}}}}^{-\frac{{q}^{2}}{4{\Delta }^{2}}}\right\rangle$$

After the further evolution from *t*_0_ to *t*_*f*_ and projecting on $|{\Psi }_{f}\rangle$, to select the cases in which the second measurement yielded the result of interest for us, we find the first measuring device to be left in the state

30 $$	\left\langle {\Psi }_{f}\right|\hat{W}({t}_{f},{t}_{0}){{{{{\rm{e}}}}}}^{-{{i}}\uplambda \hat{{A}}\hat{{p}}}\hat{W}({t}_{0},{t}_{i})\left|{\Psi }_{i}\right\rangle \left|{{{{{\mathrm{e}}}}}}^{-\frac{{q}^{2}}{4{\Delta }^{2}}}\right\rangle \\ 	 \approx \;\left\langle {\Psi }_{f}\right|\hat{W}({t}_{f},{t}_{0})(1-{{i}}\uplambda \hat{{A}}\hat{{p}})\hat{{W}}({t}_{0},{t}_{i})\left|{\Psi }_{i}\right\rangle \left|{{{{{\mathrm{e}}}}}}^{-\frac{{q}^{2}}{4{\Delta }^{2}}}\right\rangle \\ 	= \;\left\langle {\Psi }_{f}\right|\hat{W}({t}_{f},{t}_{i})\left|{\Psi }_{i}\right\rangle \left(\right.1-{{i}}\uplambda {{A}}^{{w}}\hat{{p}}\left)\right.\left|{{{{{\mathrm{e}}}}}}^{-\frac{{q}^{2}}{4{\Delta }^{2}}}\right\rangle \\ 	 \approx \;\left\langle {\Psi }_{f}\right|\hat{W}({t}_{f},{t}_{i})\left|{\Psi }_{i}\right\rangle {{{{{\mathrm{e}}}}}}^{-{i}\lambda {A}^{w}\hat{p}}\left|{{{{{\mathrm{e}}}}}}^{-\frac{{q}^{2}}{4{\Delta }^{2}}}\right\rangle \\ 	 = \;\left\langle {\Psi }_{f}\right|\hat{W}({t}_{f},{t}_{i})\left|{\Psi }_{i}\right\rangle \left|{{{{{\mathrm{e}}}}}}^{-\frac{{(q-\lambda {A}^{w})}^{2}}{4{\Delta }^{2}}}\right\rangle$$

where in the second line we have approximated the shift operator ${{{{{\rm{e}}}}}}^{-{{i}}\uplambda \hat{{A}}\hat{{p}}}$ to the first order in *λ*, in the third line we have factored out $\langle {\Psi }_{f}|\hat{W}({t}_{f},{t}_{0})\hat{W}({t}_{0},{t}_{i})|{\Psi }_{i}\rangle$, and we have used the fact that $\hat{W}({t}_{f},{t}_{0})\hat{W}({t}_{0},{t}_{i})=\hat{W}({t}_{f},{t}_{i})$ and where

31 $${A}^{w}=\frac{\left\langle {\Psi }_{f}\right|\hat{W}({t}_{f},{t}_{0})\hat{A}\hat{W}({t}_{0},{t}_{i})\left|{\Psi }_{i}\right\rangle }{\left\langle {\Psi }_{f}\right|\hat{W}({t}_{f},{t}_{i})\left|{\Psi }_{i}\right\rangle },$$

in the fourth line, we used again the first-order approximation of the shift operator and in the last line, we applied the shift operator to the state of the pointer. We have this way proved that, for small enough *λ,* the measuring device indicates the value *A*^*w*^ proving thus (20). (Note that the pre-factor $\langle {\Psi }_{f}|\hat{W}({t}_{f},{t}_{i})|{\Psi }_{i}\rangle$ is simply a normalization factor, corresponding to the probability of the second measurement to yield $|{\Psi }_{f}\rangle$.)

### Spin measurements

Let us evaluate $\left(\right.{\hat{P}}_{R}{\sigma }_{x}{\left)\right.}^{w}$, Eq. (24). To evaluate the numerator, we first note that the projector ${\hat{P}}_{R}$ measured at time *nT* + *τ* selects the wavepacket that is at this time in the right side of the box. This wavepacket originated from tunnelling in the *n* + 1 the collision. Hence

32 $${\hat{P}}_{R}\hat{U}(\tau ){\hat{U}}^{n}\left|{\uparrow }_{z}\right\rangle \big|L\big\rangle ={\cos }^{n}\epsilon \sin \epsilon \left|\tau \right\rangle$$

where $\left|\tau \right\rangle$ represents this wavepacket (see Fig. 2).

Next, the operator *σ*_*x*_ applied to the $\left|{\uparrow }_{z}\right\rangle$ spin flips it to $\left|{\downarrow }_{z}\right\rangle$, which sees the wall. The time evolution operator $\hat{U}(T-\tau )$ further propagates $\left|\tau \right\rangle$ up to the right wall, where, due to the spin being $\left|{\downarrow }_{z}\right\rangle$, it gets reflected: $\hat{U}(T-\tau )\left|\tau \right\rangle \left|{\downarrow }_{z}\right\rangle =\big|R\big\rangle \left|{\downarrow }_{z}\right\rangle$ leading to

33 $$\hat{U}(T-\tau ){\sigma }_{x}{\hat{P}}_{R}\hat{U}(\tau ){\hat{U}}^{n}\left|{\uparrow }_{z}\right\rangle \big|L\big\rangle ={\cos }^{n}\epsilon \sin \epsilon \big|R\big\rangle \left|{\downarrow }_{z}\right\rangle .$$

The evolution for the next (*N* − *n* − 1)*T*, given by ${\hat{U}}^{2N-n-1}$ can be computed immediately, using (9), and we obtain

34 $$\begin{array}{ll}&{\hat{U}}^{2N-n-1}\hat{U}(T-\tau ){\hat{P}}_{R}{\sigma }_{x}\hat{U}(\tau ){\hat{U}}^{n}\left|{\uparrow }_{z}\right\rangle \big|L\big\rangle \\ = &{\cos }^{n}\epsilon \sin \epsilon \left(\right.\cos (2N-n-1)\epsilon \big|R\big\rangle -\sin (2N-n-1)\epsilon \big|L\big\rangle \left)\right.\left|{\downarrow }_{z}\right\rangle \\ \end{array}$$

Finally, projecting on $\left|L\right\rangle \left|{\uparrow }_{x}\right\rangle$ yields

35 $$\left\langle {\uparrow }_{x}\right	|\big\langle L\big|{\hat{U}}^{2N-n-1}\hat{U}(T-\tau ){\hat{P}}_{R}{\sigma }_{x}\hat{U}(\tau ){\hat{U}}^{n}\big|L\big\rangle \left|{\uparrow }_{z}\right\rangle \\ 	 =-\displaystyle\frac{1}{\sqrt{2}}{\cos }^{n}\epsilon \sin \epsilon \sin (2N-n-1)\epsilon$$

The denominator is, using (3),

36 $$\big\langle L\big	|\left\langle {\uparrow }_{x}\right|{\hat{U}}^{2N}\left|{\uparrow }_{z}\right\rangle \big|L\big\rangle \\ 	 = \big\langle L\big|\left\langle {\uparrow }_{x}\right|{\cos }^{2N}\epsilon \big|L\big\rangle \left|{\uparrow }_{z}\right\rangle \\ 	 \quad+\,\big\langle L\big|\left\langle {\uparrow }_{x}\right|\mathop{\sum }\limits_{k=0}^{2N-1}\sin \epsilon {\cos }^{k}\epsilon \left|2N-1-k\right\rangle \left|{\uparrow }_{z}\right\rangle \\ 	 = \frac{1}{\sqrt{2}}{\cos }^{2N}\epsilon$$

From (35) and (36) we get (24):

37 $$\left(\right.{\hat{P}}_{R}{\sigma }_{x}{\left)\right.}^{w} 	= \, \frac{\big\langle L\big|\left\langle {\uparrow }_{x}\right|{\hat{U}}^{2N-n-1}\hat{U}(T-\tau ){\hat{P}}_{R}{\sigma }_{x}\hat{U}(\tau ){\hat{U}}^{n}\left|{\uparrow }_{z}\right\rangle \big|L\big\rangle }{\big\langle L\big|\left\langle {\uparrow }_{x}\right|{\hat{U}}^{2N}\left|{\uparrow }_{z}\right\rangle \big|L\big\rangle }\\ 	 = -\!\frac{{\cos }^{n}\epsilon \sin \epsilon \sin (2N-n-1)\epsilon }{{\cos }^{2N}\epsilon }$$

### Spin flux

Here we calculate the spin flux, Eq. (26):

38 $$\mathop{\sum }\limits_{n=0}^{2N-1}- \, \frac{{\cos }^{n}\epsilon \sin \epsilon \sin (2N-n-1)\epsilon }{{\cos }^{2N}\epsilon }\\ = \, -\!\sin \epsilon \mathop{\sum }\limits_{n=0}^{2N-1}\sin (2N-n-1)\epsilon +{{{{{{{\mathcal{O}}}}}}}}\left(\frac{1}{N}\right)$$

where we approximated ${\cos }^{n}\epsilon \approx 1$ for 0 ≤ *n* ≤ 2*N*. Given that $\epsilon =\frac{\pi }{2N}$ we get

39 $$\, -\sin	 \left(\frac{\pi }{2N}\right)\mathop{\sum }\limits_{n=0}^{2N-1}\sin \left((2N-n-1)\frac{\pi }{2N}\right) \\ 	= -\!\sin \left(\frac{\pi }{2N}\right)\mathop{\sum }\limits_{n=0}^{2N-1}\sin \left((n+1)\frac{\pi }{2N}\right) \\ 	= -\!\sin \left(\frac{\pi }{2N}\right)\cot \left(\frac{\pi }{4N}\right)=-2{\cos }^{2}\left(\frac{\pi }{4N}\right)\\ 	= -\!2+{{{{{{{\mathcal{O}}}}}}}}\left(\frac{1}{N}\right)$$

where we again approximated ${\cos }^{2}\left(\right.\frac{\pi }{4N}\left)\right.$ to 1 up to order 1/*N*.

## Data Availability

No data sets were generated or analyzed during the current study.
